# Preparation of Thermosensitive Fluorescent Polyacrylamide Nanofiber Membrane and Visual Temperature Sensing

**DOI:** 10.3390/polym14194238

**Published:** 2022-10-09

**Authors:** Xuejiao Tao, Zhao Dai, Yue Ma, Nan Li

**Affiliations:** 1 School of Textile Science and Engineering, Tiangong University, Tianjin 300387, China; 2 School of Chemical Engineering and Technology, Tiangong University, Tianjin 300387, China; 3 School of Chemistry, Tiangong University, Tianjin 300387, China

**Keywords:** polyacrylamide, gold nanoclusters, visual temperature sensing, fluorescent fiber

## Abstract

Fluorescent fibers are capable of discoloration behavior under special light sources, showing great potential for applications in biomedicine, environmental monitoring, heavy-metal-ion detaction, and anti-counterfeiting. In the current paper, temperature-sensitive fluorescent poly-acrylamide (PAM) nanofiber (AuNCs@PAM NF) membranes are prepared by mixing red fluorescent gold nanoclusters (AuNCs) synthesized in-house with PAM using the electrospinning technique. The AuNCs@PAM nanofibers obtained using this method present excellent morphology, and the AuNCs are uniformly dispersed in the fibers. The average diameter of the AuNCs@PAM NFs is 298 nm, and the diameter of AuNCs doped in the fibers is approximately 2.1 nm. Furthermore, the AuNCs@PAM NF films present excellent fluorescence and temperature-sensitive performance between 15 and 65 degrees. While under the 365 nm UV light source, the fiber film changes from white to red; this discoloration behavior weakens with the increase in temperature, and changes from deep to light red. Therefore, the approximate temperature can be identified using the color change, and a visual temperature-sensing effect can be achieved. The dual functions of temperature-sensitivity and fluorescent properties improve the scientificity and safety of nanofibers in the use of anti-counterfeiting technology.

## 1. Introduction

Fluorescent fiber is commonly referred to as a “functional nanometer material”; its typical feature is that it can emit a special color under irradiation of an excitation light source of a specific wavelength. When the special light source disappears, the fiber can restore its own color. Thus, to date, fluorescent fibers have attracted widespread attention based on their potential application in different fields because of their unique photochromic properties [1,2,3]. Generally, fluorescent fibers are fabricated using three common methods: melt- [4,5], wet- [6,7] and electrospinning [8,9]. Xue et al. [4] used a combination of SrAl_2_O_4_:Eu^2+^,Dy^3+^-polyethylene terephthalate (PET)/light-conversion agent-PET to prepare a luminous fiber with a skin-core structure using the melt-spinning method. The fiber can emit pure red light and has good luminous properties. Although melt-spinning is widely used due to its low cost and advanced technology, its disadvantage is the high temperature required for spinning, which restricts its application. In comparison to melt-spinning, the conditions required for wet-spinning are simple. Lu et al. [6] used the wet-spinning method to prepare fluorescent composite fibers with a mass ratio of polyvinyl alcohol (PVA)-to-hyperbranched polylysine (HBPL) of 7:1, which has good mechanical and thermal properties, and excellent fluorescence properties. However, the size of the fibers prepared by wet-spinning is large, and there are limitations to the preparation of fibers via multifunctional modification. Nevertheless, electrospinning is a powerful from of technology used for preparing functional nanofibers, which have the advantages of small size, large specific surface area, simple fabrication, and straightforward modification [10]. With the increasing interest in the subject area, many researchers have devoted their research to exploring composite fibers by co-blending polymers and other functional materials using the electrospinning method, which has led to new applications in the areas of catalysis, biology, storage, and air filtration [11,12,13]. Wang et al. [14] used SiO_2_ to coat the surface of Fe_3_O_4_ nanoparticles, and then, modified using a low-surface-energy fluorinated silane-coupling agent to prepare Fe_3_O_4_@SiO_2_@POTS. It was mixed with a PVDF solution in its preparation for spinning, and a dual-scale-structure nanofiber membrane was constructed, which improved the surface roughness of the composite membrane and presented the membrane with excellent superhydrophobicity, superparamagnetism, and tensile strength. In addition, electrospinning has been used to fabricate inorganic, organic, and hybrid (organic/inorganic) compound nanofibers with unlimited application potential [15,16]. Among them, the application of fluorescent materials in electrospinning to prepare fluorescent nanofibers has great application potential in improving anti-counterfeiting technology [9,17,18]. Shu et al. [19] prepared a polyethylene terephthalate (PET) nanoporous luminescence fiber (PNPLF) using electrospinning technology with a diameter of 250–500 nm and a pore diameter of 20–180 nm, good mechanical and luminescence properties, and fluorescent indicator functions. Fluorescence intensity changes significantly with an increase in the oil-absorption value of the fiber, which lays the foundation for its application in the field of oil absorption. Cai et al. [20] presented an effective method of synthesizing fluorescent AuNCs in situ using a BSA/PEO electrospinning fibrous membrane and described its application in the Hg^2+^-sensing process. AuNCs have attracted considerable attention from researchers [21,22] due to their good chemical stability, low biotoxicity, strong biocompatibility [23,24,25], and strong fluorescent emission. Govindaraju et al. [26] developed a one-pot synthesis method to prepare quantum-sized novel fluorescent BSA-AuNCs, and demonstrated its ability to detect L-dopamine (DA) in cerebrospinal fluid via the quenching of fluorescence, which was useful in the study of simple, less expensive, ultra-sensitive methods for the detection of DA. Uehara et al. [27] composed fluorescent gold nanoparticles and thermoresponsive polymers to prepare thermoresponsive fluorescent gold nanocomposites, and the resulting gold nanocomposites were used to stain HeLa cells.

On this basis, temperature-sensitive AuNCs were easily synthesized by using BSA as a reducing agent and stabilizer, and exhibited good fluorescence properties under a UV lamp. AuNCs@PAM NFs exhibiting fluorescence properties and temperature-sensitivity were prepared by being mixed with PAM via electrospinning, and their morphology and properties were characterized and tested. AuNCs were successfully doped into the nanofibers. AuNCs@PAM NFs presented consistent and excellent temperature-sensitive fluorescence performance with AuNCs, and the red fluorescence color gradually lightened with increasing temperature. The bifunctional nanofibers used in this study can improve the science and security of anti-counterfeiting technology, and can be broadly applied in fluorescent anti-counterfeiting technology.

## 2. Experiment

### 2.1. Preparation of AuNCs@PAM NFs

Firstly, 10 mL of 10 mM chloroauric acid (Tianjin Delan Fine Chemical Factory, Tianjin, China) solution was added to 10 mL of 50 mg/mL BSA (≥98% Sigma-Aldrich, Shanghai, China) solution, and stirred vigorously for 2 min in a water bath at 37 °C. Then, 1 mL of 1 M NaOH (Tianjin FengChuan Chemical Reagent Technology Co., Ltd., Tianjin, China) solution was added and continuously stirred for 12 h at 37 °C to produce a brown-gold nanocluster solution [28,29], which was stored at 4 °C and protected from light. BSA acted as a reducing and stabilizing agent in the reaction and limited the size of AuNCs through its own three-dimensional spatial conformation. The pH value was adjusted by NaOH, which made it possible to effectively improve the reducing ability of BSA and accelerate the reduction of Au^3+^ to Au^0^.

A total of 5 g of AuNC solution was magnetically stirred in a beaker with 14.6 g of deionized water (σ ≤ 0.1 us/cm), and 0.4 g of PAM (Mw = 5,000,000 Shanghai Macklin Biochemical Co., Ltd., Shanghai, China) particles were weighed and slowly added to the beaker, and stirred at room temperature for 14 h. AuNCs@PAM NFs were prepared using the electrospinning technique; the voltage was 13 Kv, the receiving distance was 27 cm, and the injection-pushing speed was 0.03 mm/min. The spinning was carried out at room temperature and the relative humidity was 35%.

### 2.2. Characterization

Scanning electron microscopy (SEM) and a fluorescence microscope BX53 were used to characterize the morphologies of the nanofibers. The inner lattice structure and morphologies of the AuNCs were characterized using transmission electron microscopy (TEM) (Hitachi H7650/JEM-F200, Chiyoda City, Tokyo, Japan). The diameters of the AuNCs and nanofibers were measured using Nano Measurer software. X-ray photoelectron spectroscopy (XPS, NEXSA) was performed to validate the elemental properties of the AuNCs. The crystal structure information of the AuNCs in the AuNCs@PAM NFs was measured via X-ray diffraction (XRD, D-8 ADVANC). A fluorescence spectrometer (RF-6000) was used to measure the fluorescence properties. The UV lamp model previously mentioned in this study was a tri-purpose ultraviolet analyzer (ZF-1), which uses light with a wavelength of 365 nm.

## 3. Results and Discussion

### 3.1. AuNCs

**Figure 1:**
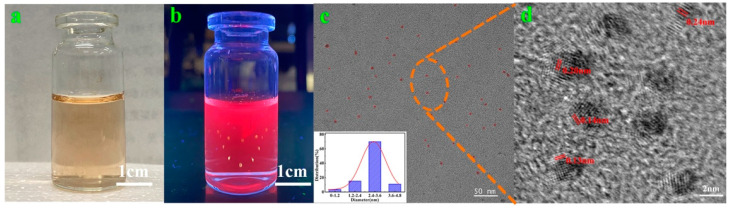
AuNCs: (**a**,**b**) appearance morphology of AuNC solution under visible light and UV lamp; (**c**,**d**) AuNC TEM (inset: AuNC diameter distribution) and HR-TEM image.

**Figure 2:**
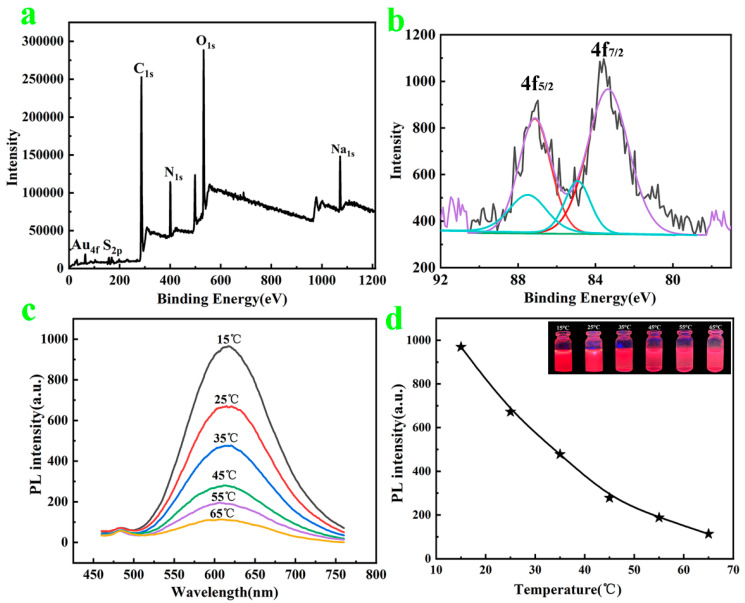
(**a**,**b**) XPS energy spectrum of AuNCs and Au4f of AuNCs; (**c**) AuNC fluorescence spectra at different temperatures; (**d**) different temperature peak-fluorescence intensities of AuNCs (inset is color change of AuNCs under UV lamp at different temperatures).

The photographs of the AuNC solution under visible light and UV lamp irradiation are presented in Figure 1a,b. The AuNC solution under visible light was brown; when the wavelength of the UV lamp irradiation reached 365 nm, the solution emitted red fluorescence characteristics. Furthermore, using rhodamine B as the standard control, the fluorescence quantum yield of the AuNCs was calculated to be 13.8% (details in S1). Figure 1c presents the TEM photographs of the AuNCs, and the morphological characteristics can be observed. The AuNCs were well-dispersed, basically spherical in shape, uniform in size, and mostly distributed at a diameter of 2–3 nm (see the diameter distribution of AuNCs provided in the inset). The lattice of the AuNCs was observed via JEM-F200 TEM, as presented in Figure 1d, and values of 0.13 nm, 0.14 nm, 0.2 nm, and 0.24 nm were obtained, corresponding to Au(111)-, (200)-, (220)-, and (311)-type face-centered cubic structure lattices, respectively; this further indicates the successful preparation of the AuNCs. To further demonstrate the AuNC elements, XPS tests were performed, as presented in Figure 2a, which exhibits absorption peaks at binding energies of 1095.08, 532.08 eV, 400.08 eV, 286.08 eV, 165 eV, and 85.08 eV, corresponding to the Na, O, N, C, S, and Au elements, respectively. As presented in Figure 2b, the binding energies of Au 4f_7/2_ of the AuNCs are deconvoluted into two distinct components centered at 83.28 and 84.88 eV, corresponding to Au^0^ and Au^+^, respectively; this indicates that Au is present in two chemical environments [28,30]. Among them, Au^0^ constitutes the gold core of the AuNCs, while Au^+^ covers the surface of the AuNCs. Taken together, the above results indicate the successful preparation of the AuNCs.

The fluorescence tests of the AuNC solution were performed at different temperatures, as illustrated in Figure 2c, to verify the temperature-sensitive properties of the successfully prepared AuNC solution. It is evident that the fluorescence intensity of the AuNC solution gradually decreases with increasing temperature (Figure 2d shows the peak-fluorescence intensity of the AuNCs at different temperatures). The fluorescence intensity is the strongest at 15 °C and the weakest at 65 °C, indicating that excellent temperature-sensitive AuNCs were prepared. The mechanism of this temperature-sensitive fluorescence followed the Boltzmann distribution: as the temperature increases, the molecular-collision frequency and nonradiative-leap rate increase, while the radiative-leap rate remains constant, thus reducing the emission intensity of the excited state (i.e., the fluorescence intensity) [31,32]. The Figure 2d inset presents the AuNC solution under a UV lamp at different temperatures; the color gradually lightens from dark red with the temperature ranging 15 °C to 65 °C, presenting characteristics that are consistent with the fluorescence spectrogram. In addition, since the fluorescence change of AuNCs is not obvious at less than 15 °C and more than 65 °C, as shown in Figures S1 and S2, it is not easy to be observe with the naked eye, and there is no great need to study it in anti-counterfeiting applications. Therefore, it was determined that a study temperature range from 15 °C to 65 °C was chosen.

### 3.2. AuNCs@PAM NFs

**Figure 3:**
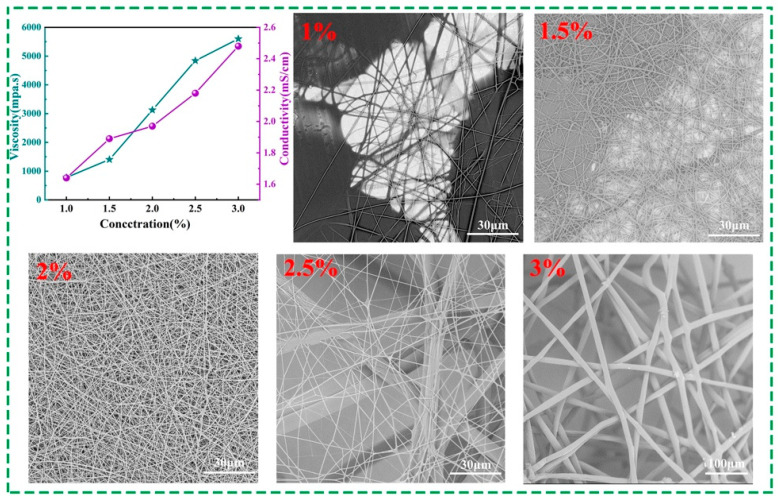
Spinning solution viscosity and conductivity at different concentrations, and the corresponding SEM images of nanofibers.

The successfully prepared AuNCs were doped in a PAM solution to prepare the electrospinning solution, and the AuNCs@PAM NFs were prepared via high-voltage electrospinning. Considering that the properties of the polymer-spinning solution play a crucial role in the morphology and performance of the nanofiber membranes, we conducted further studies on the spinning solution concentration. Figure 3 presents the viscosity and conductivity of the AuNCs@PAM-spinning solution at a room temperature of 25 °C for PAM concentrations of 1 wt%, 1.5 wt%, 2 wt%, 2.5 wt%, and 3 wt%, and for nanofibers at corresponding concentrations for SEM of their morphology. The viscosity values of the AuNCs@PAM-spinning solution at different mass fractions of PAM are presented in the green line graph, corresponding to the conductivity shown via the purple line, where both the solution viscosity and conductivity increase with the increase in the PAM mass fraction.

This may have been due to the fact that when the PAM concentration was low, the distance between the molecular bonds in the polymer PAM molecules was greater, and the intermolecular adhesion was lower. As the PAM concentration increased, the probability of the interpenetration and entanglement of the bonds between the PAM molecular chains increased, and the viscosity of the spinning solution gradually increased, too. At the same time, the conductivity of the spinning solution increased with the gradual increase in the PAM mass fraction, which was due to the fact that PAM particles are water-soluble solid materials; additionally, the conductivity in the aqueous solution increased with the increase in the concentration of the dissolved solids. The relationship between the conductivity and dissolved-solid concentration was approximated as μS/cm = 1 ppm or 2 μS/cm = 1 ppm (per million units of CaCO_3_) [33,34]. In addition, the addition of AuNCs increased the metal ions in the spinning solution, which also increased the conductivity of the spinning solution.

With a fixed spinning voltage of 13 Kv and a receiving distance of 27 cm, the nanofibers formed by solutions of different concentrations are presented in the inset of Figure 3. When the mass fraction of the solution is 1%, the spinning needle is foggy, droplets appear on the surface of the receiving membrane, and the quantity of nanofibers formed is reduced, as presented Figure 3 (1%). When the mass fraction is 1.5%, there are slightly more nanofibers on the surface of the receiving membrane. In the SEM observation of Figure 3 (1.5%), the nanofibers are uneven, presenting ribbon fibers and droplets. The reason for this is that the viscosity of the spinning solution at this concentration increased, which promoted fiber stretching and formation; however, solvent remained that was not fully evaporated, so the spinning solution jet could not be uniformly stressed into cylindrical fibers when it was stretched and formed in an electrostatic field. When the mass fraction reached 2%, the nanofibers became continuous and homogeneous, well formed, and presented a high yarn output, as shown in Figure 3 (2%). When the mass fraction was 2.5%, we observed that the nanofibers’ thickness was clearly uneven, and the thickest nanofiber reached 10 μm, as shown in Figure 3 (2.5%). When the mass fraction was increased to 3%, the nanofiber diameter drastically increased to 15 μm, and stiff fibers visible to the naked eye were formed on the surface of the receiving membrane. This was because with a further increase in the PAM concentration, the degree of entanglement between the molecules and molecular chain segments in the PAM polymer was great, resulting in the unstable operation of charged droplets in the electrostatic field and an increase in the fibers’ diameters. A comprehensive observation determined an optimal PAM concentration of 2% for the electrospinning preparation of AuNCs@PAM NFs. Additionally, the amide groups in the polymer played an important role in enhancing the fluorescence intensity of the AuNCs. According to the photographs of different concentrations of PAM solutions under a UV lamp, it can clearly be observed that the higher the polymer concentration, the better the fluorescence properties of the solution (Figure S3). The reason is that as the concentration of PAM increases, the viscosity of the spinning solution also increases, the PAM chains increase, and the chains swell with water molecules, which provide a convenient medium for particle diffusion to occur [35,36].

**Figure 4:**
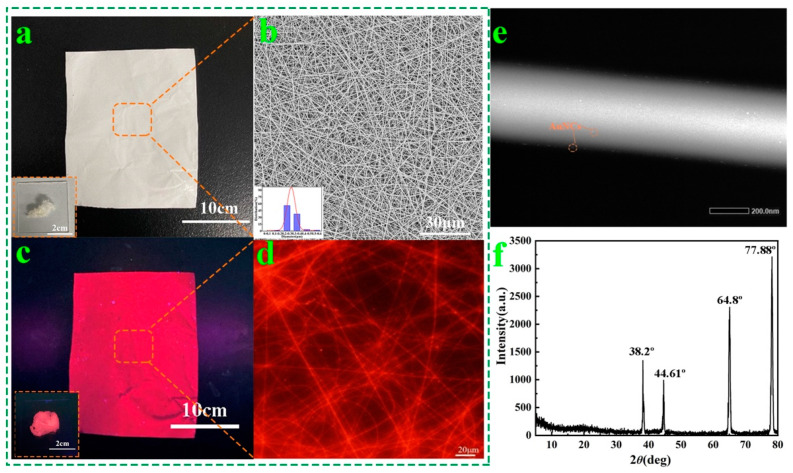
(**a**,**c**) AuNCs@PAM NFs under visible light and a UV lamp (the inset is a picture of AuNCs@PAM NFs being agglomerated); (**b**,**d**) SEM image (inset is the diameter distribution graph) and fluorescence microscopic image of AuNCs@PAM NFs; (**e**) STEM of AuNCs@PAM NF; (**f**) XRD pattern of AuNCs@PAM NFs.

The comparison photographs of AuNCs@PAM NFs prepared with a mass fraction of 2% under visible light and a UV lamp with a wavelength of 365 nm are presented in Figure 4a,c (the inset is a picture of the AuNCs@PAM NFs being agglomerated) to observe their morphology and fluorescence effect. Compared with visible light, AuNCs@PAM NFs presented red fluorescence under a UV lamp, indicating that AuNCs were well integrated in the nanofibers and exhibited excellent fluorescence properties. AuNCs@PAM NF SEM, presented in Figure 4b, shows that the fiber thickness is even; there are no obvious droplets, ribbons, or other undesirable fiber morphologies, and the average diameter is 298 nm (the inset is s diameter distribution graph). Moreover, the AuNCs@PAM NFs ere photographed using STEM-HAADF, and the STEM image (Figure 4e) clearly shows that the surface of AuNCs@PAM NFs has uniformly distributed, white, bright spots of AuNCs. The red fluorescence effect caused by the characteristic emission of AuNCs can be clearly observed by a fluorescence microscope, as shown in Figure 4d, which further proves that AuNCs are uniformly distributed in the nanofibers without agglomeration. In addition, X-ray diffraction characterization of the AuNCs@PAM NFs is shown in Figure 4f, which presents four characteristic diffraction peaks at 2θ of 38.22°, 44.61°, 64.8°, and 77.78°, corresponding to the (111), (200), (220), and (311) crystal planes of Au [30], respectively. They are consistent with the diffraction peaks and crystal structures of conventional metallic Au, further indicating that AuNCs are successfully doped in the fibers and have good crystallinity properties. In summary, the results show that AuNCs@PAM NFs were successfully prepared.

**Figure 5:**
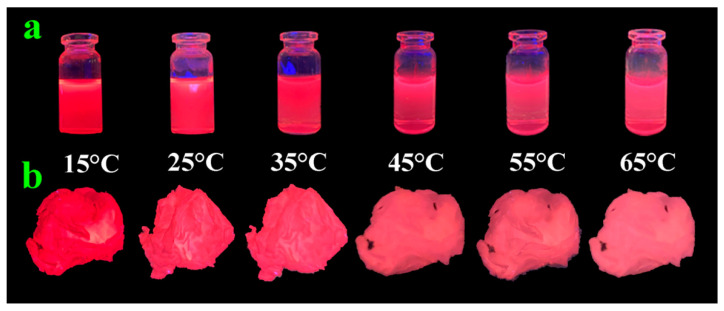
(**a**,**b**) Fluorescence change in AuNC solution and AuNCs@PAM NFs under UV lamp illumination at 15 °C–65 °C.

Figure 5a,b show the corresponding photographs of the fluorescence changes in AuNCs and AuNCs@PAM NFs under UV lamp irradiation at 15 °C–65 °C, respectively. As the temperature increased, the AuNCs and AuNCs@PAM NFs changed from dark to light red, and the fluorescence intensity decreased. The emergence of the thermosensitive behavior of the AuNCs is mainly based on the mechanism of slow, transient photoluminescence quenching; an obvious candidate was the heating of AuNCs, which led to dissipated laser power [37]. In addition, the behavioral trend of AuNCs@PAM NFs in relation to temperature was the same as that of AuNCs, which indicated that the temperature-sensitive property of AuNCs@PAM NFs was closely related to AuNCs. Moreover, the preparation of AuNCs@PAM NFs by co-blending AuNCs with PAM did not destroy the original property of the AuNCs. Similarly, the results also show that our prepared AuNCs@PAM NFs had an excellent temperature-sensitivity properties, which lays a certain foundation for anti-counterfeiting research on fluorescent fibers. In addition, the repeatability test results of the thermosensitive fluorescence properties of AuNCs@PAM NFs are presented in Figure S4. It is clearly presented that AuNCs@PAM NFs have good fluorescence-recovery performance and stability.

### 3.3. Application

**Figure 6:**
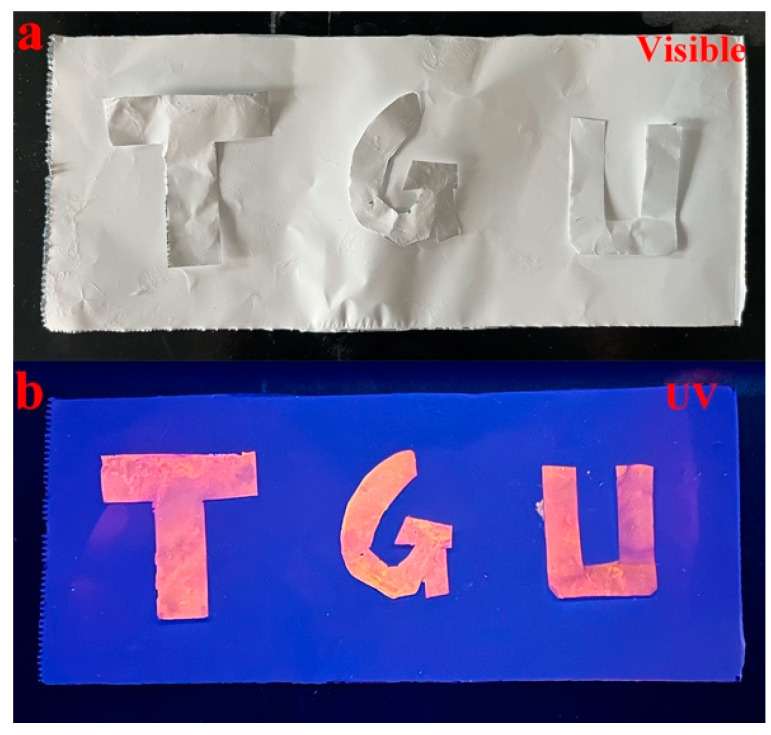
(**a**,**b**) Removed AuNCs@PAM NF “TGU” letters and underlying PAM NFs under visible light and a UV lamp.

The letters “TGU” were removed from the prepared AuNCs@PAM NFs, placed on the surface of the PAM NFs, and observed under visible light and a UV lamp, as shown in Figure 6a,b. Under the visible light, the “TGU” appeared the same as the underlying PAM NFs, but under the UV lamp, it could be observed that the red fluorescent letters significantly differed from the color of the PAM NFs. This indicates that AuNCs@PAM NFs have potential value in practical anti-counterfeiting applications.

## 4. Conclusions

In this paper, temperature-sensitive AuNCs with a diameter of about 2.1 nm were easily fabricated using BSA as a reducing agent and stabilizer, and presented excellent fluorescence properties under a UV lamp. The red fluorescence color of the AuNCs was deepest at 15 °C, and the color gradually decreased with increasing temperature. By doping AuNCs into PAM, AuNCs@PAM NFs were successfully prepared via the traditional electrospinning technique with an average diameter of 298 nm, and were characterized using TEM, SEM, XRD, fluorescence microscopy, and UV. AuNCs@PAM NFs presented excellent temperature-sensitivity properties and an obvious fluorescence effect under a UV lamp, indicating their potential for the application of fluorescent fibers in heating stickers, temperature-sensitive responses, and anti-counterfeiting materials.

## Data Availability

The data presented in this study are available on request from the corresponding author.

## Supplementary Materials

The following supporting information can be downloaded at: https://www.mdpi.com/article/10.3390/polym14194238/s1, S1. Fluorescence QY calculation of AuNCs; Figure S1. AuNCs solution at 5 °C and 15 °C under UV lamp illumination; Figure S2. AuNCs solution at 65 °C and 75 °C under UV lamp illumination; Figure S3. The photos of different concentrations of PAM solutions under UV lamp; Figure S4. Plots of fluorescence intensities of AuNCs@PAM NF at 620 nm vs. number of heating cycle days. Reference [38] is cited in the supplementary materials.
