# Supplementary material for: Preparation of Thermosensitive Fluorescent Polyacrylamide Nanofiber Membrane and Visual Temperature Sensing
Source: Polymers (Basel). 2022 Oct 9;14(19):4238. doi: 10.3390/polym14194238 (PMC9571245; doi:10.3390/polym14194238)
Supplement: Supplementary file 1 [file polymers-14-04238-s001.zip › polymers-1918645-supplementary.pdf]

Supplementary Materials

Preparation of Thermosensitive Fluorescent Polyacrylamide Nanofiber Membrane and Visual Temperature Sensing

Xuejiao Tao [1], Zhao Dai [2,*], Yue Ma [2] and Nan Li [3]

[1] School of Textile Science and Engineering, Tiangong University, Tianjin 300387, China
[2] School of Chemical Engineering and Technology, Tiangong University, Tianjin 300387, China
[3] School of Chemistry, Tiangong University, Tianjin 300387, China
* Correspondence: daizhao@tiangong.edu.cn; Tel: +86-83955621; Fax: +86-22-83955451

S1: Fluorescence QY calculation of AuNCs

Using rhodamine B as the standard control [R1], the fluorescence QY of AuNCs was calculated according to the following formula:

$$Q_C = Q_R * \frac{I_C}{I_R} * \frac{A_R}{A_C} * \frac{\eta_C^2}{\eta_R^2} \qquad \text{(S1)}$$

where "Q" is the quantum yield, "I" is the integral fluorescence intensity, "η" is the refractive index of the solvent, and "A" is the absorbance. Subscripts "C" and "R" represent AuNCs and rhodamine B, respectively.

And the fluorescence QY of AuNCs was calculated to be 13.8% using Equation (S1).

R1. Yue, J.; Yu, L.; Li, L.; Liu, P.; Mei, Q.; Dong, W.F.; Yang, R. One-Step Synthesis of Green Fluorescent Carbon Dots for Chloride Detecting and for Bioimaging. *Front Chem* **2021**, *9*.

S2:

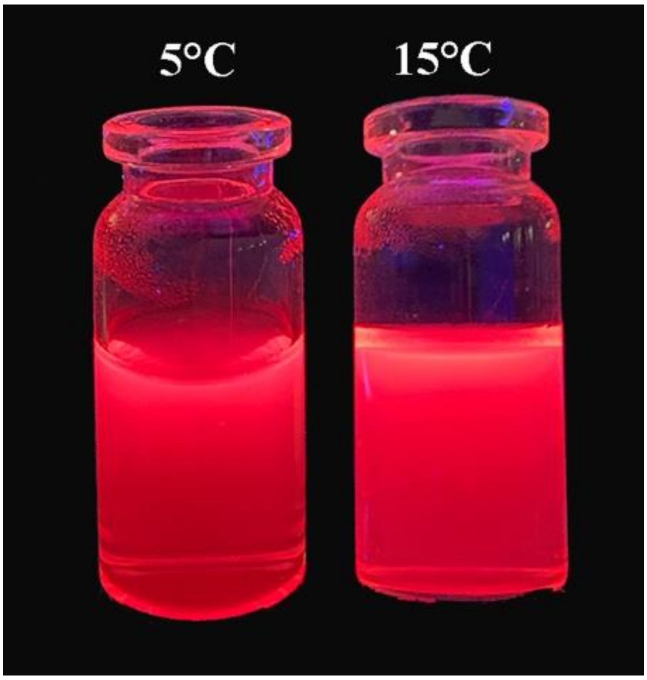

Figure S1. AuNCs solution at 5°C and 15°C under UV lamp illumination.

S3:

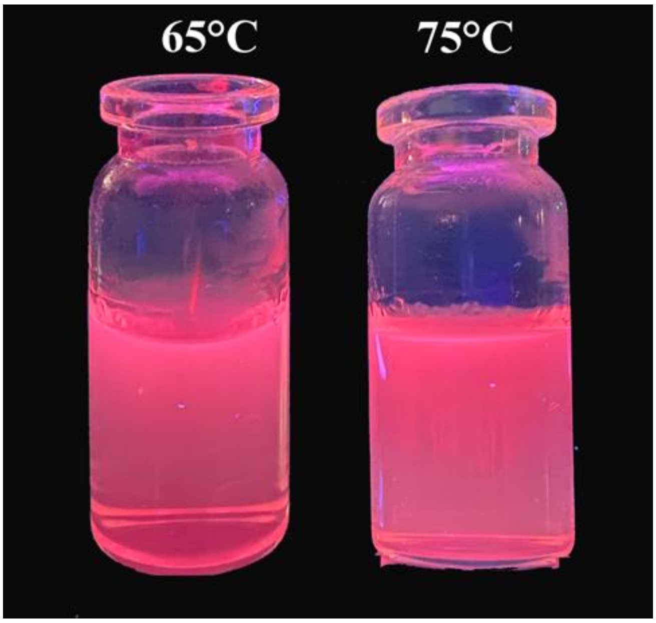

Figure S2. AuNCs solution at 65°C and 75°C under UV lamp illumination.

S4:

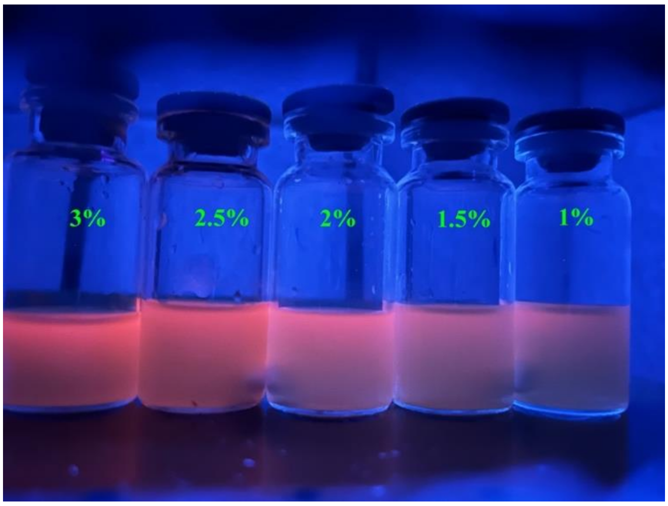

Figure S3. The photos of different concentrations of PAM solutions under UV lamp.

S5:

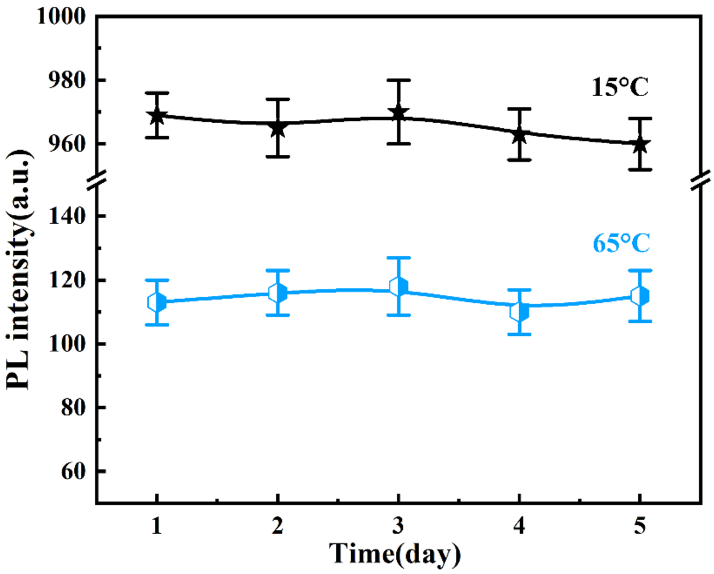

Figure S4. Plots of fluorescence intensities of AuNCs@PAM NF at 620 nm vs. number of heating cycle days.
